# Integrating Psychosocial Risks With Emerging Evidence for Borderline Personality Disorders in Adolescence: An Update for Clinicians

**DOI:** 10.7759/cureus.40295

**Published:** 2023-06-12

**Authors:** Nihit Gupta, Mayank Gupta, Jayakrishna S Madabushi, Faiza Zubiar

**Affiliations:** 1 Psychiatry, University of West Virginia, Glen Dale, USA; 2 Psychiatry and Behavioral Sciences, Southwood Psychiatric Hospital, Pittsburgh , USA; 3 Psychiatry, Alabama College of Osteopathic Medicine, Birmingham, USA; 4 Psychiatry, The Trenton Psychiatric Hospital, Trenton, USA

**Keywords:** adolescence, trauma, psychosocial factors, maternal factor, bullying, family environment, abuse, borderline personality disorder

## Abstract

Borderline personality disorder (BPD) has seen significant advances in the knowledge of its developmental phenomenology during late childhood and adolescence. Various genetic, neurobiological, psychological, and social factors are implicated in the etiology of BPD. With emerging evidence on BPD development in adolescence, the review focused on recent literature to understand the role of psychosocial risk factors. The effects of adverse familial environment, physical, emotional, verbal, and sexual abuse, intergenerational transmission of psychopathological traits, maternal neglect and rejection, low socioeconomic status, bullying victimization, and dating violence were reviewed to understand their role in the development of BPD.

BPD is a highly complex, serious, and enduring mental illness that has now been widely accepted to have symptoms that onset in early adolescence and could be diagnosed as early as age 12. BPD symptoms are stable, phenomenologically distinct from externalizing and internalizing disorders, and often present with co-occurring disorders, which during assessment could not explain impairments associated with BPD. New measures like the Difficulties in Emotion Regulation Scale (DERS), detailed developmental histories, understanding of psychosocial risks, shared decision-making, and psychoeducation could assist in early diagnosis and improvement of long-term outcomes.

The implementation of evidence-based treatments is a challenge given higher costs and access to services; therefore, modifications in the treatment based on the core principles of these strategies should be considered. It is imperative to screen for psychosocial factors early in higher-risk groups. The assessment of familial factors, parental histories of psychopathologies, and histories of childhood abuse is important in context with impairing symptoms of clinical presentation and dimensional aspects of self-functioning. The role of family therapies, parental psychoeducation, and the integration of trauma-informed care approaches are important for clinical outcomes. Also, coordinated efforts with multiple stakeholders like school awareness programs, anti-bullying policies, legislation, and enforcement of existing laws might be instrumental in addressing issues related to victimization by peers.

## Introduction and background

BPD is a prevalent (0.9%-3% in the adolescent population [1-3]) complex disorder with a multifactorial etiology, including genetics, maladaptive attachment, and trauma, that cannot be explained by a normative developmental perspective or externalizing and internalizing disorders. It also subsumes theories derived from descriptive analytical literature, attachment theories, temperamental studies, self-psychology, identity formation, and empirical knowledge of its links with co-occurring disorders, impairments, costs, long-term prognosis, and lastly, the emergence of evidence-based interventions.

Besides variable prevalence and gender differences, individuals with BPD are overrepresented in clinical settings and psychiatric-referred populations. BPD is present in up to 20% of people in outpatient psychiatric care [4,5] and up to 50% of people receiving inpatient psychiatric treatment [6]. With associated stigma, clinicians’ countertransference, and risks related to implicit impulsivity, BPD has been a controversial diagnosis with poor reliability and many ranks of misinformation and bias. Adolescence is a transition period characterized by significant neurodevelopmental changes; it is also associated with mood swings, impulsivity, and emotional disturbances, and thus many clinicians remain hesitant to diagnose BPD in adolescents [7-11].

Since the Diagnostic and Statistical Manual of Mental Disorders, Third Edition (DSM-III), the diagnosis has been allowed for adolescents, but there is both hesitancy and misinformation that continue to prevail to date, even though in the last decade many traditional propositions related to adolescent BPD have been replaced by strong empirical studies in groundbreaking, cutting-edge research. The DSM permits clinical diagnosis when symptoms and impairments have been present for more than 12 months [12]. The presence and diagnosis of BPD in individuals aged < 18 years are now accepted by international organizations and classification systems, including the United Kingdom’s National Institute for Clinical Excellence (NICE) [13], the Australian National Health and Medical Research Council (NHMRC) Guidelines, and the American Psychiatric Association (APA) [14].

In clinical practice, with the increase in the global burden of mental illness among children and adolescents, it is critical to identify these individuals early and provide awareness and treatment opportunities. However, there are many clinical caveats. The psychiatrically referred adolescents with BPD have previously been diagnosed with externalizing and internalizing disorders, which (on many occasions) both individuals and families consider could better explain BPD symptomatology but are not open to exploring other dimensional aspects of personality. Given the traditional view, adolescence is a turbulent stage with higher emotional drive, and BPD traits are normative and not stable enough to establish a clinical diagnosis. Therefore, in the absence of any biomarkers, genetic tests, or other objective signs or symptoms, there are serious challenges in time-limited real-world clinical encounters to reliably establish a BPD diagnosis. The etiological understanding of the development of BPD remains poorly established [15,16]. A biopsychosocial model has been proposed for BPD development, which emphasizes the involvement of various genetic, neurobiological, biochemical, familial, behavioral, and social factors in BPD development.

The twin studies conducted on adolescents provided evidence for the heritable nature of BPD [17,18]. Its association with genes encoding for the serotonin transporter (5HTT) [19, 20], tryptophan hydroxylase [21], 5‐HT2a [22], 5HT2c [23], or monoamine oxidase A [24] has been reported in adults and adolescents. Additionally, structural changes in the fronto‐limbic region [25] and prolonged activation of the hypothalamic-pituitary-adrenocortical (HPA) axis have been reported in adolescents [26]. There has been emerging evidence to establish a neurobiological basis for BPD with unclear epigenetic pathways; however, interventions to address modifiable psychosocial risks are likely to provide better outcomes [27] .

Due to these serious clinical challenges, in this review, we explored a question to identify specific psychosocial and social risks associated with BPD in adolescence. In clinical settings, this information is most readily available to the providers, and identifying these risks in addition to the developmental history could assist in clinical diagnostics. The primary objective of this review is to identify if BPD development in adolescents has a relationship with specific risk factors and could be utilized for early diagnosis and interventions.

Methods

**Figure 1:**
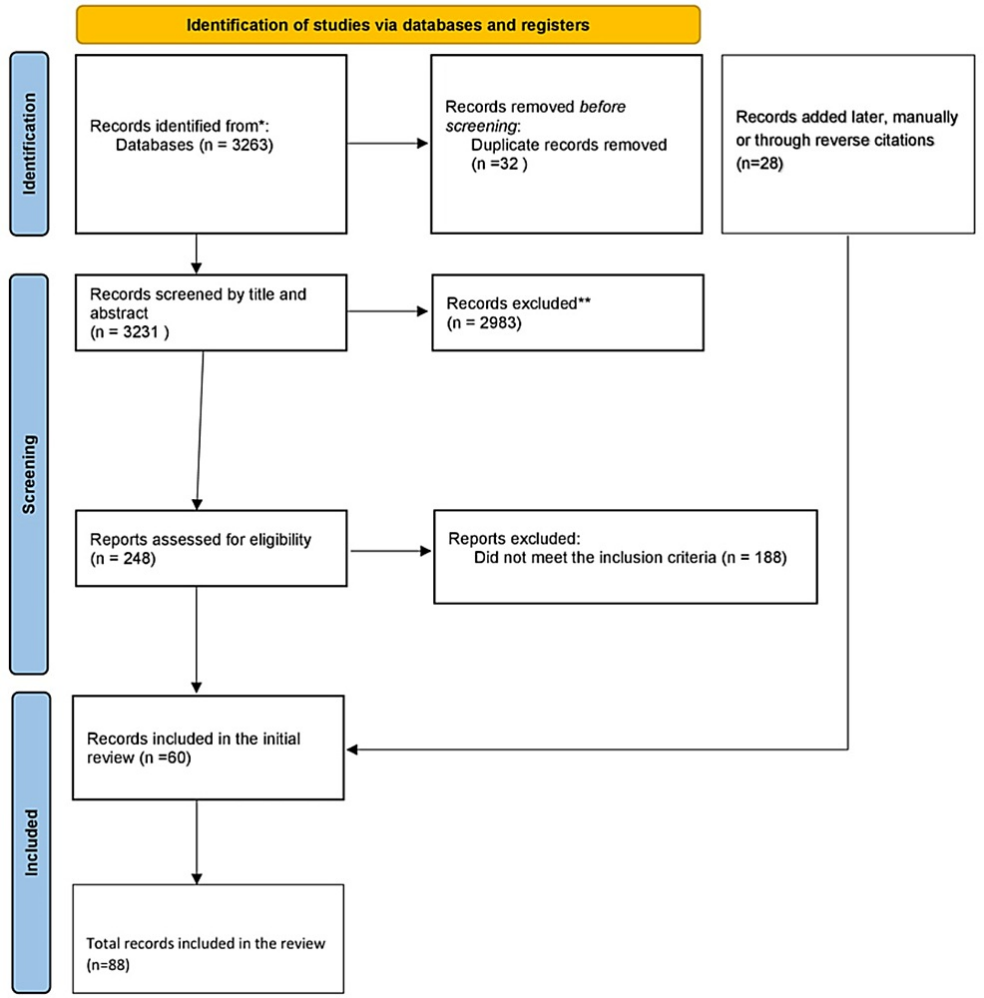
PRISMA protocol PRISMA: Preferred Reporting Items for Systematic Reviews and Meta-Analyses

Searches were conducted in PubMed, PsychINFO, and Google Scholar databases to identify studies reporting BPD in adolescents (aged under 18 years) published between inception and January 2023. It was intended that the search criteria be broad in order to identify all articles on "borderline personality disorder," along with age filters so as to identify all articles between the ages of 0 and 18 years in the language English. The search was conducted from the date of inception of each database to the date of the search. We identified 3231 articles after removing duplicates with automated software. After reviewing the titles and abstracts of the 3231 articles identified, we selected 248 studies that met our inclusion criteria and explored the psychological and social factors associated with developing BPD in children and adolescents, with particular attention paid to studies evaluating the impact of family factors, trauma, and abuse on BPD development. BPD studies focusing exclusively on adults, genetic and neurobiological factors, and BPD treatment were excluded. For our initial manuscript, we used 60 studies from the 248 that were identified. A total of 28 articles were added later, both manually and through reverse citation. The Preferred Reporting Items for Systematic Reviews and Meta-Analyses (PRISMA) guidelines were followed and adhered to (Figure 1).

## Review

We reviewed recent empirical studies on the implications of various psychosocial factors in BPD development in subjects aged < 18 years. Particularly, the study presents an overview of several longitudinal, retrospective, and cross-sectional studies conducted on inpatients, outpatients, and the general population (aged < 18 years) from worldwide demographic areas. These studies used DSM‐IV or DSM‐V criteria for BPD evaluation. Few studies assessed parents and their children, especially the ones evaluating the transgenerational effects of BPD. Table 1 presents the salient characteristics of the studies reviewed. This review succinctly overviews and highlights the etiological role of various psychological factors, including familial adversities, childhood abuse, parental psychopathology, peer victimization, and others, in BPD development during adolescence.

**Table 1:** Key points of the studies reviewed

Key points:
The clinical diagnosis of borderline personality disorder in individuals aged ≤ 18 years is now clinically acceptable.
It is important to assess for adverse familial environment, maladaptive parenting, parental psychopathology, parental divorce, parental hostility, attachment disorganization, childhood neglect, inter-parental conflicts, alcohol and drug abuse, and delinquency.
Parent-child relationship problems, parental suicide, low socioeconomic status, exposure to intimate partner violence (IPV), childhood abuse, bullying, dating victimization violence, and sexual orientation act as strong risk factors for BPD development during adolescence.
A detailed assessment of psychosocial factors will be helpful in making a formal clinical diagnosis and individualizing treatment strategies.

Familial risks

In the last two decades, several studies have explored the association between familial adversities and BPD development. These studies identified maladaptive parenting, parental psychopathology, domestic violence, parental divorce, low socioeconomic status (SES), parental hostility, overprotective and rejecting parenting styles, and attachment disorganization as strong predictors of BPD symptoms [15,28-31].

In a community-based longitudinal study (N = 738), Johnson et al. highlighted the impact of childhood neglect, specifically emotional, physical, and supervision neglect, on the development and severity of PDs in adolescence and early adulthood [32]. Likewise, Winsper et al. found that children exposed to adverse family environments, suboptimal parenting methods, and interparental conflicts displayed a higher risk of the early development of BPD symptoms, particularly at the age of 11 [33]. Guile et al.'s retrospective chart review study reported that several familial factors, particularly the parental history of psychological disorder, alcohol/drug abuse, and delinquency, witnessing parental disagreement, disturbed parent-child relationships, and having siblings instead of being a single child, exhibited significant associations with BPD traits [34]. Similarly, a higher risk of BPD development was reported in female adolescents exposed to family adversities like poverty, single-parent households, and difficult life circumstances [35]. These findings highlight the role of familial factors in the development and severity of BPD symptoms.

Importantly, maternal psychopathology played a central role in the precocious onset of BPD [29,36,37]. Herr et al. reported a strong correlation between maternal BPD symptoms and interpersonal functioning, depressive symptoms, and attachment cognition (insecure attachment) in offspring [36]. Similar observations were reported by Reinelt and Aldinger. The study reported longitudinal transmission of borderline traits from mothers to children, mediated via maladaptive mother-child interactions [29]. These results were in concordance with the findings of Barnow et al., which showed that maternal BPD symptoms could predict the severity of the offspring’s psychopathology [37]. Interestingly, Infurna et al. reported a significant association between paternal psychiatric symptoms and adolescent BPD. Additionally, a link between the transgenerational effect of parental bonding experiences and BPD development in offspring was also reported. [38]. The transgenerational effect of the parental experience of childhood adversities on BPD development was further established in another study [39]. Kurdziel et al. reported higher incidences of physical abuse, emotional abuse, and neglect in patients with BPD mothers. Severity, chronicity, and involvement in multiple subtypes of abuse were associated with BPD symptoms in adolescents [40]. Kors et al. reported a higher frequency of intergenerational transmission of child maltreatment in families with a maternal history of BPD, where dysregulated interpersonal relationships played a pivotal role [41]. These effects of parental BPD on the psychopathology of offspring might be attributed to a maladaptive family environment, dysfunctional parenting styles, low parental affection, aversive parental behavior, overprotection, and maternal rejection [28,29,38,39].

Additionally, recent evidence suggests the role of low SES and intimate partner violence (IPV) in BPD development in adolescents. Children exposed to violence between their caregivers are at higher risk of developing emotional or behavioral problems [42,43]. In a recent longitudinal study, Sharp et al. reported high incidences of BPD in adolescents exposed to IPV. Interestingly, these adolescents showed a deviation from the normative decline pattern usually observed in BPD adolescents, wherein BPD symptoms increase during adolescence, followed by a decline thereafter into adulthood [31]. Thus, the study highlighted the need to develop and utilize parent-focused IPV programs to minimize negative outcomes for offspring. In a recent study, Moscoso et al. reported a higher frequency of parental suicide attempts in BPD patients, which also correlated with the severity of BPD symptoms [30].

Several studies have identified low SES as a risk factor for the development of several mental and physical disorders. Cohen et al. identified low SES as a predictor of BPD in early teens [44]. Similar results were reported by Crawford et al. [45]. The study also reported a severe impact of early, extended separation from the mother before the age of five years.

Children born into families with low SES, parental history of psychopathologies, and higher stress levels often experience interfamilial conflict, improper housing conditions, an unstable family structure, and parental unemployment [46,47]. Therefore, exposure to such adverse conditions might have a detrimental effect on an individual’s interpersonal skills, identity development, decision-making abilities, and emotional regulation [48]. All these findings highlight the need for a detailed evaluation of familial factors and parental histories of psychological disorders. Additionally, both parents should be involved in clinical evaluation and treatment. Key findings can be reviewed in Table 2.

**Table 2:** Familial adversities as a risk factor for BPD development

Author, Year	Key findings
Johnson et al., 2000 [32]	Childhood neglect has an impact on the development and severity of personality disorders.
Winsper et al., 2012 [33]	Children exposed to suboptimal parenting and interparental conflicts are at a higher risk of early BPD.
Guilé et al., 2016 [34]	Parental psychological disorders and substance use are associated with the development of BPD.
Stepp et al., 2016 [35]	Familial adversaries can increase the risk of BPD, especially among females.
Herr et al., 2008 [36], Barnow et al., 2013 [37]	Longitudinal transmission of borderline traits via maladaptive mother-child interaction has been reported.
Infurna et al., 2016 [38], Reichl et al., 2017 [39], Kurdziel et al., 2018 [40], Kors et al., 2020 [41]	There could be a link between the transgenerational effect of the parental bonding experience and BPD development in the offspring.
Evans et al., 2008 [45], Vu et al., 2016 [43], Cohen et al., 2008 [44], Crawford et al., 2009 [45]	Low SES and IPV could also have a role in the development of BPD.

Trauma and its association with BPD in adolescence

In the last few decades, childhood abuse has emerged as an important predictor for the development of personality disorders (PDs), especially BPD. Childhood abuse is known to be associated with higher levels of dependency, violent and impulsive behavior, suicidality, substance misuse, and borderline traits [49]. Interestingly, individuals with a history of childhood abuse are four times more likely to develop PDs during adulthood [50].

Linehan’s biosocial theory of BPD development highlighted the impact of an adverse childhood environment on BPD development [51,52]. Merza et al. reported a higher prevalence of adverse childhood experiences (ACE), namely neglect, emotional, physical, and sexual abuse, or witnessing trauma, in BPD inpatients. A higher incidence of severe sexual abuse, involving incest, penetration, and repetitive abuse, was reported in BPD patients. Sexual abuse, intrafamilial physical abuse, and neglect by caretakers acted as the strongest predictors of BPD [53]. Similar findings were reported by Infurna et al., wherein sexual abuse, general family functioning, and low maternal care acted as specific and independent predictors of adolescent BPD [54]. Bounoua et al. identified emotional abuse as an important contributor to BPD development [55]. Turniansky et al. assessed female inpatients and reported more severe clinical characteristics in patients with a history of childhood sexual abuse [56].

Hecht et al. reported an increased incidence of BPD symptoms in individuals subjected to physical abuse and neglect [57]. In contrast to an old study [50], this study reported no association between BPD symptoms and sexual abuse. Temes et al. reported verbal abuse by caregivers to be common in BPD adolescents [58]. These results were in concordance with the findings of Johnson et al. [59]. In a community-based longitudinal study, Jovev et al. reported childhood neglect to be a strong predictor of BPD symptoms during adolescence [60].

Although we currently have a limited understanding of the relationship between the two, BPD is known to be characterized by a higher frequency of non-suicidal self-injury (NSSI) events and suicide attempts. Kaplan et al. reported a higher frequency of NSSI in BPD adolescents with a history of abuse prior to admission to an intensive treatment program involving dialectical behavior therapy (DBT). Additionally, a history of childhood abuse was associated with a five-fold higher rate of lifetime suicide attempts [61]. Hessel et al. evaluated the impact of childhood abuse on BPD development in adolescents with NSSI disorder (N=152). Individuals exposed to childhood adversities displayed higher BPD symptoms [62]. Key findings can be reviewed in Table 3.

**Table 3:** Childhood abuse and BPD in adolescents

Author, Year	Key findings
Salsman & Linehan, 2012 [51], Crowell SE, Beauchaine TP, Linehan MM, 2009 [52]	Adverse childhood experiences (ACE) have an impact on the development of BPD.
Salsman & Linehan, 2012 [51], Crowell SE, Beauchaine TP, Linehan MM, 2009 [52], Merza et al., 2015 [53], Infurna et al., 2016 [54]	ACE, which includes emotional abuse, caretaker neglect, intrafamilial physical abuse, severe sexual abuse, and repetitive abuse, can be an important contributor to BPD development.
Kaplan et al., 2016 [61]	NSSIs are more frequent among individuals with BPD with a history of abuse. Additionally, a history of abuse is associated with a five-times higher risk of a lifetime suicide attempt.

Social factors in BPD development

Bullying Victimization

Bullying victimization by peers is a repetitive and systematic abuse of power that includes physical, verbal, psychological, and cyber aggression [63,64]. Bullying has been associated with the development of personality disorders [65-67] and neurobiological alterations in the brain [68]. Wolke et al. reported that peer victimization, overt or relational, contributed to the early onset of BPD symptoms at the age of 11.8 years [69]. Similar results were reported by Winsper et al. [15]. Antila et al. assessed psychiatric inpatients and reported a four-fold increased risk of PD development only in female subjects exposed to bullying during childhood and adolescence, indicating a gender-specific association [66]. Laporte et al. reported that victims of physical abuse by peers displayed a significant predisposition toward BPD development [70]. In a recent study in outpatient settings, Bozatello et al. identified emotional abuse, bully victimization, the presence of alcohol or drug abusers in the household, and physical neglect as important predictors for the early onset of BPD [71].

All these studies indicate a strong association between bullying victimization by peers and BPD development during late childhood or adolescence. Teachers, parents, and clinicians must address bullying immediately, especially among females [72]. Anti-bullying programs and policies at the school level [73,74] and strict execution of anti-bullying laws can be particularly helpful [75-78].

Dating Violence

The majority of research evidence has established a strong correlation between attachment insecurity and BPD development. During adolescence, dating relationships could have serious implications for the psychopathology of BPD [79]. Reuters et al. (2015) reported a positive correlation between BPD symptoms and dating violence victimization (DVV), especially among females [80]. Similar findings were reported by Hatkevich et al., wherein studied adolescent inpatients with co-occurrence of BPD features and DVV displayed a higher risk of self-harming behavior [81]. In another longitudinal study, Vanwoerden et al. reported a link between BPD traits and victimization in dating relationships in females. Among males, DVV, particularly psychological violence (which includes emotional and verbal abuse), acted as a serious risk factor for BPD development [82]. All these findings highlight the role of DVV in borderline pathology and also suggest incorporating awareness and education about DVV into school-level intervention programs.

Sexual Minority

Limited evidence is available for an association between sexual orientation and BPD in adolescents. Reuter et al. studied an ethnically diverse sample of 835 adolescents, and the sexual minority teens were overrepresented in the BPD group and scored higher on borderline features. The study also highlighted the importance of implementing intervention programs for psychosocial outcomes for sexual minority youth [83]. Social factors implicated in BPD can be reviewed below in Table 4.

**Table 4:** The implication of social factors in BPD development

Author, Year	Key findings
Antila et al., 2017 [66], Wolke et al., 2012 [69], Laporte et al., 2012 [70], Bozzatello et al., 2020 [71], Gupta et al., 2021 [72]	Peer victimization, including bullying, physical abuse, and emotional abuse, contributed to the early onset of BPD symptoms.
Reuter et al., 2015 [80], Vanwoerden et al., 2019 [82]	Positive correlation between BPD symptoms and DVV, especially among females but also among males.
Reuter et al., 2016 [83]	Sexual minority teens were overrepresented in the BPD group and scored higher on borderline features.

Clinical implications in identifying risks specific to adolescent BPD

This overview suggests screening for specific psychosocial risk factors in children and adolescents at higher risk of developing BPD. These measures would aid in the early identification of BPD, which is particularly important to avoid misdiagnosis and provide timely treatment [34]. It’s imperative to assess individuals’ familial environment and parental history of psychopathology. Family therapy-based programs also target alleviating parental psychopathology, which may address familial transmission and diminish the risk of impairment in offspring. Family therapy particularly aims at improving parent-child relationships and creating a suitable family environment [36,38,84]. In patients with BPD and any coexistent abuse, the clinical interventions may include strategies to help recognize maladaptive thoughts, feelings, and behaviors arising due to abuse [59,62]. Particularly, trauma-informed care (TIC) approaches are highly recommended to be integrated into the treatment program. TIC approaches are focused on understanding, recognizing, and alleviating trauma-related symptoms and post-traumatic stress in adolescents [85,86]. The involvement of parents during therapy sessions might also assist in receiving desired outcomes. Parental psychoeducation is particularly important to increase their awareness of the illness. Parents must be trained in behavior management and effective communication [2]. Since peer victimization has serious psychological implications, parents and teachers must address bullying and DVV during adolescence. The association of childhood abuse and peer victimization with suicide ideation and NSSI necessitates trauma-based therapies to alleviate posttraumatic stress and reduce the risk of such behaviors [81]. Therefore, a deeper understanding of the implications of psychosocial risk factors in BPD development will help unravel multifactorial etiological mechanisms and the course of the illness. Key findings of studies reporting psychosocial factors and their role in early diagnosis and treatment can be reviewed in Table 5.

**Table 5:** Role of psychosocial factors in early diagnosis and treatment

Author, Year	Key findings
Eyden et al., 2016 [84]	Family therapy-based programs have been shown to diminish the risk of impairment in offspring. Parental training in behavioral management and effective communication can be helpful.
Flavin et al., 2022 [85], Bryson et al., 2017 [86]	Trauma-informed care (TIC) is highly recommended and found to be effective.

Discussion

The review focuses on identifying specific psychosocial risks associated with adolescent BPD, which could provide a framework to establish early diagnostics and assist clinicians working with individuals with BPD in developing contextual formulations. Although it’s evident from the results that there are limits to the complete contextual framework about the development of BPD in early adolescence, as an alternative vantage point, together with these findings’ amalgamation with some major developments in the last decade in the empirical literature, this is a leap forward in the understanding of BPD. The newer research has addressed some critical questions regarding BPD development during adolescence.

It was also accepted that although personality is a stable, ego-syntonic construct, it is "under construction" in adolescence. The traits are seen as early as infancy and have been extensively studied in myriad temperamental profiles, and the famous Thomas and Chess "Goodness-to-Fit" paradigm underscores the role of environmental factors. Personality development encompasses overlapping constructs like early temperamental styles, self-concept, and identity formation within a healthy scaffolding environment to establish stable, coherent capacities to manage internal needs in the realities of the world.

The BPD traits are stable irrespective of the developmental changes, with symptoms distinct from those of other externalizing or internalizing disorders. There are even studies on preschoolers to understand the risks related to internalizing and externalizing psychopathology, high adverse childhood experiences (ACEs), and early suicidality with BPD [87]. The presence of internalizing and externalizing disorders at an earlier age adds to the risks, and the chronology of these disorders often predates the onset of BPD and not the other way around [88, 89]. These developmental phenotypes of BPD are often observed in clinical practice, which often adds to the clinical challenges and thereby needs correlation with specific risks. When in doubt, measurement with established tools could be an effective strategy. Yet, the risk factors are commonly associated with many other illnesses, and with the transdiagnostic symptomology of BPD, it’s difficult to recognize their relationship to specific risks that could be the target of therapeutics. The Difficulties in Emotion Regulation Scale (DERS) has been established as a reliable tool with good construct validity and predictive validity in adolescents [90]. Its use has been largely in academic settings, but it is also highly effective in measuring the response to interventions and taking steps toward measurement-based care.

Advances in adolescent BPD knowledge led to a traditional DSM-5 categorical nosology for PD with the emergence of "Another Model for Personality Disorders" (AMPD) [91]. These underscore the dimensional aspect of BPD, which is qualitatively distinct from externalizing and internalizing disorders. In psychoanalytic traditions, BPD is understood in terms of a poorly integrated or diffused self and a chronic vulnerable state manifested by interpersonal stress. This also integrates Peter Blos's ideas about the "second individuation" process that occurs during adolescence and attempts by adolescents to develop a coherent identity [92].

There are significantly higher rates of cooccurring disorders with BPD, which adds to the clinical conundrums and uncertainty with diagnostics given the risks of affecting therapeutic alliance due to the stigma associated with BPD [93]. However, when emerging empirical research is integrated, it paves the way for the early diagnosis and treatment of BPD. Therefore, understanding the specific risks is paramount to providing context and developing shared decision-making with individuals with BPD. Without reconciliation of early trauma, maladaptive attachment, and understanding of BPD as a distinct stable entity different from externalizing or internalizing disorders that emerge in early adolescence, the effectiveness of the psychiatric formulations and treatment planning may not yield desired outcomes.

Lastly, there have been ongoing debates about BPD's association with non-suicidal self-injury (NSSI), which often co-occurs with BPD and has an onset between ages 12 and 14. While more research is needed to establish a better understanding of its developmental phenomenology, there have been ongoing discussions about even considering NSSI as an exclusive diagnostic category [94].

The multimodal treatment plan, which optimizes the treatment of concurring conditions while addressing the core deficits of BPD with psychotherapeutic interventions like DBT, mentalization-based therapy (MBT), etc., may be more appropriate. Diagnostic overshadowing is not uncommon, given that affective psychopathology, emotional reactivity or dysregulation, and impulsivity coexist with BPD and may delay diagnosis and treatment. With higher costs and a lack of access to DBT, MBT, transference-focused therapy (TFP), and other evidence-based interventions, there have been many recent efforts to develop models to incorporate these principles when working with individuals with BPD.

## Conclusions

BPD is a highly complex, serious, and enduring mental illness that has now been widely accepted to have symptoms that onset in early adolescence. BPD symptoms are stable, phenomenologically distinct from externalizing and internalizing disorders, and could be reliably diagnosed clinically as early as age 12, and the use of objective measures improves diagnostic validity when transdiagnostic symptoms of the cooccurring disorders obscure phenotypic presentations. It is critical to incorporate the last decade of compelling empirical evidence to understand the role of highly specific risk factors that point towards plausible epigenetic interplay in BPD development in children and adolescents. It is widely accepted that BPD has a multifactorial etiology, and with more acceptance of this seriously impairing diagnosis at an earlier age, there is a need for more research-based evidence to assist in diagnosis and treatment. The evidence is counterintuitive and suggests a more detailed psychosocial assessment and an alternative understanding of BPD in dimensional terms. These details are crucial for formal clinical diagnoses and individualized treatment strategies. Awareness of these developments would assist clinicians, families, schools, children's and youth social services, and policymakers in mitigating the negative outcomes associated with this disorder.
